# Treatment attrition rates and relevant risk factors in multiple myeloma: A real-world study in China

**DOI:** 10.3389/fphar.2023.979111

**Published:** 2023-01-12

**Authors:** Wenjiao Tang, Jinrong Yang, Yan Li, Li Zhang, He Li, Jie Wang, Yi Liao, Chunlan Zhang, Ying Qu, Yuhuan Zheng, Ting Niu

**Affiliations:** Department of Hematology, Institute of Hematology, West China Hospital Sichuan University, Chengdu, China

**Keywords:** multiple myeloma, attrition rate, line of therapy, frontline treatment, transplant

## Abstract

**Background:** For multiple myeloma (MM), the proportions of patients reaching the subsequent line of therapy (LOT) decline gradually and real-world data describing the attrition rates of LOT in Chinese MM were limited. Herein, we investigated the attrition rates by subsequent LOTs and their relevant risk factors in MM patients in China.

**Methods:** MM patients who had been hospitalized and received at least one LOT from January 2008 to August 2019 in West China Hospital Sichuan University were retrospectively recruited. Demographic and clinical characteristic data were obtained from the “HemaTank” Chinese Multiple Myeloma Database. The Cox proportional hazards regression model was applied to analyze the risk factors of frontline treatment attrition.

**Results:** A total of 1,255 newly diagnosed MM were enrolled, with 573 (45.7%) patients receiving only one LOT and 682 (54.3%) patients receiving more than one LOT. Thalidomide with dexamethasone/prednisone was the most common frontline treatment before 2017, while bortezomib-based regimens constituted the majority of frontline treatment in 2017 and beyond. The attrition rates from the first to the fifth LOT exhibited a gradual upward trend (45.7%, 48.7%, 58.9% and 62.5%, respectively). Meanwhile, 54.3%, 27.9%, 11.5%, and 4.3% of all the enrolled MM patients received a second, third, fourth and fifth LOT. MM who underwent autologous stem cell transplantation (ASCT) showed lower attrition rates across all LOTs (range 12%–56.8%) than MM without ASCT (range 49.1%–64.5%). The multivariate Cox regression model revealed that ISS stage III (HR 2.07, *p* < .001), elevated LDH (HR 1.47, *p* = .006), and comorbidities such as amyloidosis (HR 1.63, *p* = 0 .01), hepatic disease (HR 1.36, *p* = .022), pulmonary disease (HR 1.38, *p* = .022), and cardiac disease (HR 1.62, *p* = .004) were independent risk factors for MM patients attritted from the frontline treatment.

**Conclusion:** In this study, the attrition rates were generally high and increased gradually across all LOTs. Nearly half of MM patients received only one LOT, and higher tumor burden and more comorbidities may be associated with fewer subsequent LOTs. The high attrition rates highlight the importance of applying the most optimal frontline treatment regimen rather than salvaging subsequent LOTs.

## Introduction

Multiple myeloma (MM) is the second most common hematologic malignancy, which is a biologically heterogeneous disease characterized by clonal evolution during disease progression and remains incurable currently (Morgan et al., 2012; Rajkumar, 2020; Yang et al., 2022). The proportions of MM patients reaching the subsequent line of therapy (LOT) decline gradually (Raab et al., 2016; Yong et al., 2016). Besides, the treatment response rate, response duration, overall survival (OS) and progression-free survival (PFS) decrease with each successive LOT in MM, while the incidence of toxicity increases (Yong et al., 2016; Cook et al., 2018; Hajek et al., 2018; Verelst et al., 2018). The response depth and duration of the frontline treatment of MM are associated with PFS and OS (Landgren and Iskander, 2017; Yan et al., 2019). Therefore, an optimal frontline treatment regimen rather than salvaging subsequent LOTs is critical for improving the outcomes of MM. A good understanding of the attrition rates of LOTs and risk factors of the frontline treatment attrition is essential to provide evidence for choosing the most optimal frontline therapy and clinical treatment sequence among the available standard of care in the real-world practice.

Attrition rates between LOTs vary in regions but are commonly high in MM (Fonseca et al., 2021). A real-world study across Europe demonstrated that the attrition rates of MM patients gradually increased from the first to fifth LOT (36.1%, 43.2%, 73.2%, and 72.8%, respectively) (Yong et al., 2016). Findings derived from 5704 MM patients in the United States (US) indicated that attrition rates are 50.4% between the first and second LOT and 54.5% between the second and third LOT (MacEwan et al., 2018). Meanwhile, Fonseca et al. observed that the attrition rates across all LOTs for non-transplant MM patients are higher than for transplant patients based on the data identified from three US patient-level databases (Fonseca et al., 2020). Over the past decades, the treatment pattern of MM in China has significantly changed with the availability of more novel agents (Huang et al., 2017; Cai et al., 2022). However, the treatment regimens during the same period are clinically distinct between China and European countries and the United States countries in real-world practice due to the later launch and medical insurance coverage of new agents in China (Qian et al., 2018; Fonseca et al., 2020; He et al., 2020; He et al., 2021; Atrash et al., 2022). Therefore, the attrition rates of LOTs may vary due to the different frontline treatment patterns in China. Besides, multiple factors may influence the selection of the frontline regimens for MM patients, such as clinical characteristics, performance status, comorbidities and economic burden, resulting in potential risk factors of treatment attrition.

To the best of our knowledge, there were no studies on attrition rates of distinct LOTs and their relevant risk factors in China. In this study, we aim to elucidate the attrition rates of separate LOTs and their associated risk factors in Chinese MM patients by collecting real-world data from the West China Hospital Sichuan University (WCHSCU). Findings gleaned from this work would provide valuable clues for MM enrollment in clinical trials and patient management in clinical practice.

## Materials and methods

### Study population

Newly diagnosed MM patients from January 2008 to August 2019 in WCHSCU were retrospectively enrolled in this study. The cutoff date of follow-up data was 31 October 2021. MM patients included in this study must meet the following criteria: 1) the diagnosis of MM was confirmed to the criteria of the International Myeloma Working Group (Rajkumar et al., 2014), 2) newly diagnosed MM patients who have been hospitalized in the department of hematology in WCHSCU, and 3) receiving at least one LOT. Exclusion criteria included patients who received only short-term glucocorticoid therapies and smoldering MM or monoclonal gammopathy of undetermined significance. This study was approved by the Ethics Committee of WCHSCU and conducted according to the Declaration of Helsinki.

### Data extraction

Data were obtained from the “HemaTank” Chinese Multiple Myeloma Database (HCMMD). “HemaTank” was a project that aimed to construct databases that collect and analyze the real-world data of patients with multiple myeloma based on a standard dataset (Niu et al., 2022), and now it has been expanded to 19 tertiary referral hospitals in China. The baseline demographic features (e.g., age and gender), laboratory examination indexes (e.g., anemia, thrombocytopenia, leukocytopenia, renal insufficiency, hypercalcemia, immunoparesis, and ISS stage) and comorbidities (e.g., tuberculosis, hypertension, diabetes, amyloidosis, hepatic disease, pulmonary disease, and cardiac disease) were extracted from the database. Treatment regimens across all LOTs extracted from the database were manually reviewed. The common treatment regimens include thalidomide-dexamethasone/prednisone (TD/TP), bortezomib-dexamethasone (BD), bortezomib-cyclophosphamide-dexamethasone (BCD), bortezomib-thalidomide-dexamethasone (BTD), vincristine-adriamycin-dexamethasone-thalidomide (VADT), melphalan-prednisone-thalidomide (MPT), cyclophosphamide-dexamethasone/prednisone (CD/CP), and cyclophosphamide-thalidomide-dexamethasone (CTD).

### Evaluation of attrition rates and associated risk factors

One LOT was defined as an initial administration of at least one kind of anti-tumor drug until an unplanned addition of a new drug or switching to a different drug or combination of new drugs due to any reason such as disease progression, lack of response, inadequate response, toxicity or other reasons (Rajkumar et al., 2015). In order to elucidate the proportion of MM patients dropped from each LOT, we calculated the attrition rates between distinct LOTs. Duration of treatment (defined as from the start time of treatment to the end of a specific LOT) was evaluated for each LOT. In this study, the attrition rate was defined as the ratio of MM patients who did not have a record of a subsequent LOT owing to death, loss to follow-up, or still stayed at the current LOT (i.e., no subsequent treatment during the study period). To understand the attrition rates in distinct clinical settings, we conducted the subgroup analysis by using the variables of age, gender, autologous stem cell transplantation (ASCT), treatment regimen and time. To elucidate the potential clinical and biological factors that influenced MM patients to receive more LOTs, we partitioned patients into 1 LOT (only treated with one LOT) and >1 LOT subgroups and examined the distinct distribution of the potential factors between two subgroups.

### Statistical analysis

R software (version 4.1.2) was employed to achieve relevant analyses and plots. The continuous and categorical variables of the two LOT subgroups were assessed with Wilcoxon rank-sum test (Wilcoxon test) and Chi-square test, respectively. Exploration of factors associated with attrition rates was based on Cox proportional hazards regression model, and in the outcome binary variable, “1” indicates patient attrition, and “0” indicates non-attrition. Two-sided *p* values less than .05 were considered to be statistically significant.

## Results

### Baseline characteristics of MM

Finally, a total of 6,665 diagnosed MM patients were collected from the HCMMD of WCHSCU, including 3,175 hospitalized MM patients. Among them, 1,255 newly diagnosed MM patients meeting the inclusion criteria were enrolled in the study. The detailed demographic information, baseline clinical characteristics and comorbidities were presented in Table 1. The median age of all the enrolled MM patients was 62, ranging from 19 to 91. Only 117 (9.3%) patients received an ASCT treatment. Amongst, 682 (45.7%) patients were treated with only one LOT, and the residual patients (54.3%) received more than one LOTs. Their distinct distributions of baseline characteristics between subgroups were illustrated in Table 1. MM patients receiving more than one LOTs were significantly younger than patients with only one LOT (median age: 61 vs. 63 years, *p* = .001). A significant higher proportion of ISS stage III were observed in 1 LOT subgroup than >1 LOT subgroup (34.5% vs. 51.5%, *p* < .001). Besides, MM patients in the> 1 LOT subgroup harbored a significantly lower proportion of anemia, thrombocytopenia, renal insufficiency and comorbidities such as amyloidosis, hepatic diseases, pulmonary diseases and cardiac disorders (Table 1).

**TABLE 1:** Clinical features of included 1255 MM patients and their distinct distribution between patients with 1 LOT and >1 LOT.

Characteristics	Sample, n	>1 LOT (*n* = 682)	1 LOT (*n* = 573)	*p* values
Age (years) range, median	19–91, 62	19–91, 61	25–86, 63	.001
Age (years)				
<65	761	438 (57.6)	323 (42.4)	.005
≥65	494	244 (49.4)	250 (50.6)	
Sex				
Male	727	407 (56.0)	320 (44.0)	.171
Female	528	275 (52.1)	253 (47.9)	
ISS stage				
I	323	225 (70.0)	98 (30.0)	<.001
II	348	213 (61.2)	135 (38.8)	
III	478	231 (48.3)	247 (51.7)	
NA	106			
ASCT				
Yes	117	103 (88.0)	14 (12.0)	<.001
No	1,138	579 (50.9)	559 (49.1)	
Lactic dehydrogenase				
Low (<250)	1,009	580 (57.5)	429 (42.5)	<.001
High (≥250)	232	100 (43.1)	132 (56.9)	
NA	14			
Anemia				
Yes	673	326 (48.4)	347 (51.6)	<.001
No	575	356 (62.0)	219 (38.0)	
NA	7			
Leukocytopenia				
Yes	323	180 (55.7)	143 (44.3)	.651
No	925	502 (54.3)	423 (45.7)	
NA	7			
Thrombocytopenia				
Yes	281	134 (47.7)	147 (52.3)	.008
No	967	548 (56.7)	419 (43.3)	
NA	7			
Hypercalcemia				
Yes	94	47 (50.0)	47 (50.0)	.339
No	1,145	631 (55.1)	514 (44.9)	
NA	16			
Immunoparesis				
Full immunoparesis	826	463 (56.1)	363 (43.9)	.094
Partial immunoparesis	224	129 (57.6)	95 (42.4)	
No immunoparesis	117	78 (66.7)	39 (33.3)	
NA	88			
Renal insufficiency				
Yes	243	98 (40.3)	145 (59.7)	<.001
No	1,004	583 (58.1)	421 (41.9)	
NA	8			
Tuberculosis				
Yes	34	20 (58.8)	14 (41.2)	.595
No	1,221	662 (54.2)	559 (45.8)	
Hypertension				
Yes	299	150 (50.2)	149 (49.8)	.097
No	956	532 (55.6)	424 (44.4)	
Diabetes mellitus				
Yes	123	62 (50.4)	61 (49.6)	.356
No	1,132	620 (54.8)	512 (45.2)	
Amyloidosis				
Yes	119	42 (35.3)	77 (64.7)	<.001
No	1,136	640 (56.3)	496 (43.7)	
Hepatic diseases				
Yes	230	103 (44.8)	127 (55.2)	.001
No	1,025	579 (56.5)	446 (43.5)	
Pulmonary diseases				
Yes	591	270 (45.7)	321 (54.3)	<.001
No	664	412 (62.0)	252 (38.0)	
Cardiac disorders				
Yes	135	46 (34.1)	89 (65.9)	<.001
No	1,120	636 (56.8)	484 (43.2)	

### Frontline treatment patterns in MM

**FIGURE 1:**
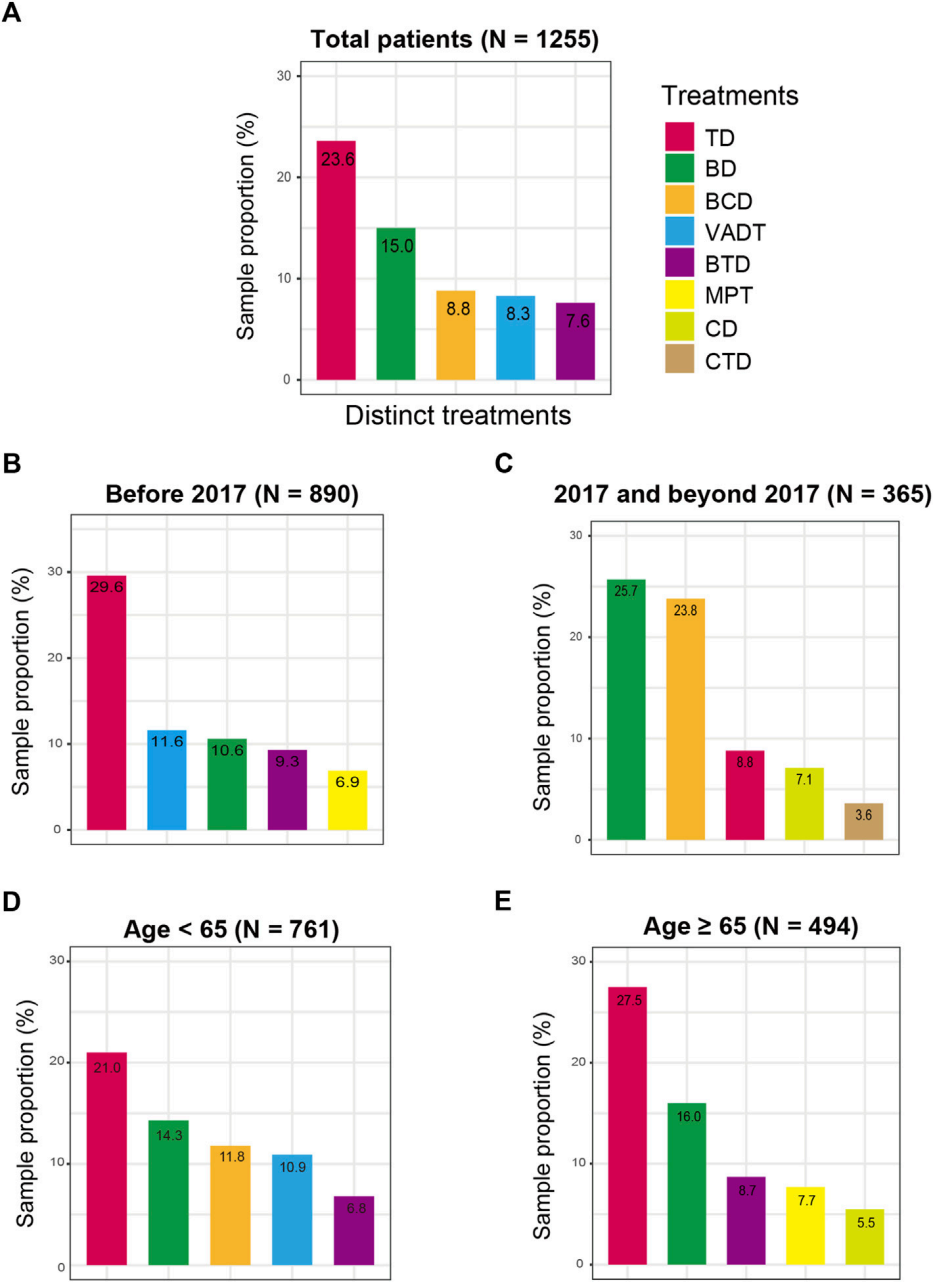
Frontline treatment patterns for MM patients in distinct clinical subgroups. **(A)** in the total MM patients; **(B)** before 2017; **(C)** after 2017; **(D)** in MM patients aged less than 65 years old; **(E)** in MM patients age 65 years or older.

Distinct chemotherapy agents are used for the frontline treatment of MM patients, and we therefore investigated the common therapy drugs and patterns in the MM first LOT. In overall patients, results indicated that TD (23.6%) was the most frequently used treatment pattern in Chinese MM frontline clinical settings (Figure 1A), followed by BD (15.0%), BCD (8.8%), VADT (8.3%), and BTD (7.6%). Considering that bortezomib was not included in National Reimbursement Drug List (NRDL) list until 2017, we also explored the evolution of treatment patterns for patients with MM before 2017 or 2017 and beyond. Subgroup analysis of frontline treatment demonstrated that the main treatment patterns before 2017 (*N* = 890) included TD (29.6%), VADT (11.6%), BD (10.6%), BTD (9.3%), and MPT (6.9%) (Figure 1B). Nevertheless, among MM patients in 2017 and beyond (*N* = 365), BD (25.7%) was the most commonly applied therapeutic regimen, followed by BCD (23.8%) and TD (8.8%) (Figure 1C). In addition, the choice of the frontline treatment patterns was usually influenced by the performance status and health conditions, so we performed the subgroup analyses of treatment regimens by age. Among patients <65 years old, the main treatment patterns were TD (21.0%), BD (14.3%), BCD (11.8%), VADT (10.9%), and BTD (6.8%), while TD (27.5%), BD (16.0%), BTD (8.7%), MPT (7.7%), and CD (5.5%) were frequently employed among patients ≥65 years old (Figures 1D, E).

### Treatment duration between distinct LOTs

**FIGURE 2:**
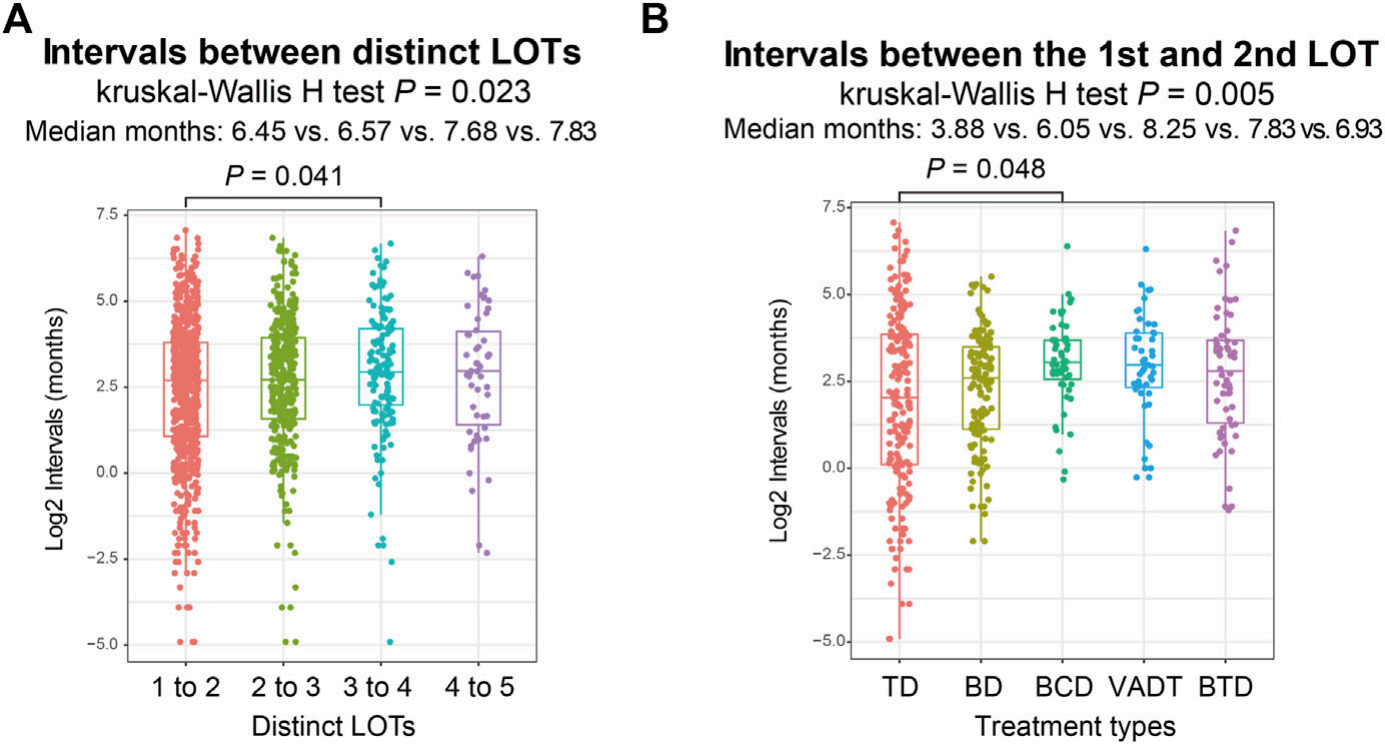
**(A)** Intervals between distinct LOTs in total MM patients. **(B)** Intervals from the first LOT to the second LOT among different frontline treatment regimens.

We calculated the treatment duration between distinct LOTs to understand the intervals between each LOT. Among the 1,255 patients, the median treatment duration for the 1st to 2nd LOT, 2nd to 3rd LOT, 3rd to 4th LOT and 4th to 5th LOT were 6.45, 6.57, 7.68, and 7.83 months respectively (Figure 2A). Further comparisons revealed a significant difference between the treatment duration of 1st to 2nd LOT and 3rd to 4th LOT (Wilcoxon rank-sum test *p* = .041; Figure 2A). We then investigated the distinct frontline treatment durations for the commonly used five treatment regimens (i.e., TD, BD, BCD, VADT, and BTD), which were significantly different (median time: 3.88, 6.05, 8.25, 7.83, and 6.93 months; Kruskal-Wallis H test *p* = .005; Figure 2B), with TD exhibiting the shortest treatment duration and BCD treatment showing the longest treatment duration (Wilcoxon rank-sum test *p* = .048; Figure 2B).

### Attrition rates of subsequent LOTs

**FIGURE 3:**
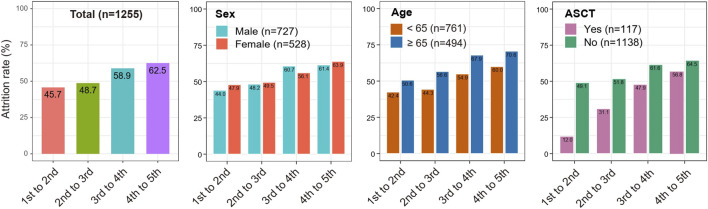
The detailed attrition rate between distinct LOTs in specific clinical subgroups.

**FIGURE 4:**
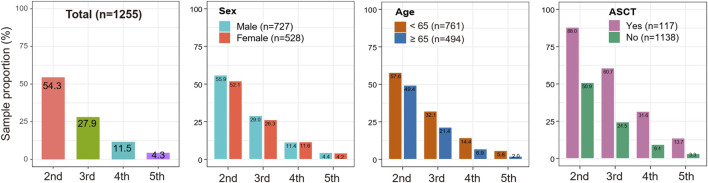
Proportions of MM patients reaching subsequent LOTs under distinct clinical subgroups.

We calculated distinct attrition rates with the increased numbers of LOT. Results from the total MM patients revealed that the attrition rates from the first to the fifth LOT exhibited a gradual upward trend (45.7%, 48.7%, 58.9% and 62.5% for the 1st to 2nd LOT, 2nd to 3rd LOT, 3rd to 4th LOT and 4th to 5th LOT, respectively). (Table 2; Figure 3). As a result, 54.3%, 27.9%, 11.5%, and as few as 4.3% of all the MM patients enrolled in the study received a second, third, fourth and fifth LOT respectively (Figure 4).

**TABLE 2:** Attrition rates between distinct LOTs in specific clinical settings.

Group	Attrition rate, n (%)			
	1st to 2nd LOT	2nd to 3rd LOT	3rd to 4th LOT	4th to 5th LOT
**Total (*N* = 1,255)**	573 (45.7)	332 (48.7)	206 (58.9)	90 (62.5)
Sex				
Male (*N* = 727)	320 (44.0)	196 (48.2)	128 (60.7)	51 (61.4)
Female (*N* = 528)	253 (47.9)	136 (49.5)	78 (56.1)	39 (63.9)
Age				
<65 (*N* = 761)	323 (42.4)	194 (44.3)	134 (54.9)	66 (60.0)
≥65 (*N* = 494)	250 (50.6)	138 (56.6)	72 (67.9)	24 (70.6)
ASCT				
Yes (*N* = 117)	14 (12.0)	32 (31.1)	34 (47.9)	21 (56.8)
No (*N* = 1,138)	559 (49.1)	300 (51.8)	127 (61.6)	69 (64.5)

Taking into account that clinical factors potentially influence MM attrition rate, we then performed stratification analyses based on the variables of gender, age, and ASCT. As shown in Figure 3, consistent trends of elevated attrition rates were observed in gender, age and ASCT subgroup analysis. Meanwhile, MM patients ≥65 years old showed relatively higher attrition rates than patients <65 years old in correspondingly LOTs (50.6% vs. 42.4%, 56.6% vs. 44.3%, 67.9% vs. 54.9% and 70.6% vs. 60.0% for the 1st to 2nd LOT, 2nd to 3rd LOT, 3rd to 4th LOT and 4th to 5th LOT, respectively). Similarly, MM who underwent ASCT showed higher attrition rates across all LOTs (range 12%–56.8%) than MM without ASCT (range 49.1%–64.5%). Notably, 13.7% of transplant MM patients received the fifth LOT, while only 3.3% of non-transplant MM patients entered the fifth LOT (Figure 4).

### Risk factors associated with the treatment attrition

The incidence of MM patients reaching the second LOT during follow up in Supplementary Figure S1. To elucidate possible factors that are linked with subsequent MM LOTs, we collected multi-dimension indexes and compared their differences between 1 LOT (*N* = 573) and >1 LOT (*N* = 682) subgroups under univariate and multivariate Cox regression models (Table 3). Univariate analysis demonstrated that higher age, ISS stage III, full immunoparesis, anemia, thrombocytopenia, renal insufficiency, hypercalcemia, lactic dehydrogenase, hypertension, diabetes, amyloidosis, hepatic disease, pulmonary disease, and cardiac disease exhibited significant risk factors for MM patients attrited from the frontline therapy (all *p* < .05). Further multivariate Cox regression analysis revealed that ISS stage III, elevated lactic dehydrogenase, amyloidosis, hepatic disease, pulmonary disease, and cardiac disease remained still significant after adjusting for multiple confounding factors (all adjusted *p* < .05). Aa a result, patients in ISS III stage (HR: 2.07, 95% CI: 1.42–3.01, *p* < .001), with high lactic dehydrogenase (HR: 1.47, 95% CI: 1.11–1.94, *p* = .006), amyloidosis (HR: 1.63, 95% CI: 1.13–2.36, *p* = .01), hepatic disease (HR: 1.36, 95% CI: 1.05–1.77, *p* = .022), pulmonary disease (HR: 1.38, 95% CI: 1.08–1.76, *p* = .022), and cardiac disease (HR: 1.62, 95% CI: 1.16–2.26, *p* = .004) were more likely to be attrited (Table 3).

**TABLE 3:** Univaraite and multivaraite Cox regression models regarding factors associated with attrition rates.

Variables	Univaraite HR (95% CI)	*p* values	Multivaraite HR (95% CI)	*p* values
Age	1.01 (1.00–1.02)	.002	1.00 (.99–1.02)	.429
Sex				
Male	References			
Female	1.05 (.89–1.24)	.582		
ISS stage				
I	References		References	
II	1.36 (1.05–1.76)	.021	1.27 (.89–1.81)	.18
III	2.62 (2.07–3.32)	<.001	2.07 (1.42–3.01)	<.001
Immunoparesis				
No immunoparesis	References		References	
Partial immunoparesis	1.03 (.71–1.49)	.886	1.02 (.53–1.28)	.458
Full immunoparesis	1.40 (1.01–1.95)	.044	1.28 (.85–1.46)	.216
Anemia				
No	References		References	
Yes	1.70 (1.44–2.02)	<.001	1.16 (.88–1.52)	.297
Leukocytopenia				
No	References			
Yes	1.01 (.84–1.22)	.932		
Thrombocytopenia				
No	References		References	
Yes	1.36 (1.13–1.64)	.001	.88 (.67–1.16)	.374
Renal insufficiency				
No	References		References	
Yes	2.13 (1.76–2.58)	<.001	1.28 (.94–1.74)	.124
Hypercalcemia				
No	References		References	
Yes	1.75 (1.29–2.37)	<.001	1.16 (.76–1.78)	.479
Lactic dehydrogenase				
<250	References		References	
≥250	1.60 (1.31–1.94)	<.001	1.47 (1.11–1.94)	.006
Tuberculosis				
No	References			
Yes	.99 (.58–1.69)	.999		
Hypertension				
No	References		References	
Yes	1.27 (1.06–1.54)	.011	.94 (.72–1.24)	.673
Diabetes				
No	References		References	
Yes	1.39 (1.07–1.82)	.015	1.17 (.81–1.69)	.413
Amyloidosis				
No	References		References	
Yes	1.68 (1.32–2.14)	<.001	1.63 (1.13–2.36)	.01
Hepatic disease				
No	References		References	
Yes	1.45 (1.19–1.77)	<.001	1.36 (1.05–1.77)	.022
Pulmonary disease				
No	References		References	
Yes	2.12 (1.79–2.51)	<.001	1.38 (1.08–1.76)	.022
Cardiac disease				
No	References		References	
Yes	2.21 (1.76–2.78)	<.001	1.62 (1.16–2.26)	.004

## Discussion

In this study, we identified the commonly applied frontline treatment pattern and assessed the attrition rates of distinct LOTs by collecting and analyzing the data of 1255 MM Chinese patients derived from the HCMMD. Notably, risk factors for the frontline treatment attrition were also explored. To the best of our knowledge, this is the first study to assess the attrition rates of LOTs for Chinese MM patients based on the real-world data. Besides, this study analyzed the frontline treatment patterns based on the largest sample size in China. Findings from this work would provide theoretical evidence for MM patients to receive distinct therapies at specific clinical stages.

This study demonstrated that MM frontline therapies in China are distinct from US and Western countries. The thalidomide-containing regimen is the most common frontline treatment in all the MM patients enrolled in the study, while Bortezomib-containing and lenalidomide regimens are the primary frontline treatment among MM patients from US and Western countries in the same period (Fonseca et al., 2020; He et al., 2021; Kumar et al., 2021; Atrash et al., 2022). It may be partly attributed to the economic factor and the availability of new agents. According to the MM dataset in this study, we also observed the obvious transitions of treatment patterns over time. A significant change in the treatment pattern of MM patients occurred around 2017, after which Bortezomib-based regimens became the main protocol. Although bortezomib was approved by China Food and Drug Administration in 2005, it was not included in the Chinese NRDL list until 2017. It can be seen that whether it can be reimbursed by medical insurance in China is a very critical factor influencing the treatment choices of physicians and patients in real-world practice. We also noticed that age might be a potential influence factor for MM patients to receive distinct frontline chemotherapies, and the treatment pattern of older MM was slightly different from other studies (Chan et al., 2019; Al Saleh et al., 2021; Cejalvo et al., 2021; Jimenez-Zepeda et al., 2021). TD, BD, and BTD were frequent treatment regimens in both age subgroups. Besides, BCD and VADT were also observed in the frontline treatment among patients <65 years old, but MPT and CD treatments were usually applied among patients ≥65 years old, which may be explained that oral administration of less intensive chemotherapy regimens is more convenient and safer for older adults.

This study demonstrated that the attrition rates were generally high in Chinese MM patients, almost consistent with previous studies in other countries (Raab et al., 2016; Yong et al., 2016; MacEwan et al., 2018; Antunes et al., 2019; Fonseca et al., 2020; Mohyuddin et al., 2021). The attrition rates fluctuated from 45.7% to 62.5% across all LOTs and exhibited a gradually increased tendency with the increase of LOT. Nevertheless, the increasing trend of attrition rates may be attributed to worse treatment efficacy, more comorbidities, death, or family economic burden with the subsequent LOT. As a result, 54.3%, 27.9%, 11.5%, and as few as 4.3% of all the MM patients enrolled in the study received a second, third, fourth and fifth LOT. Similarly, approximate 47%, 27%, 16%, and 9% of MM patients received a second, third, fourth and fifth LOT based on the data derived from three databases in the United States (up to 2018) (Fonseca et al., 2020), and 32%–16%, 14%–38%, 15%, and 1% of European MM patients received a second, third, fourth, and fifth LOT (Raab et al., 2016; Yong et al., 2016; Antunes et al., 2019). Therefore, when MM patients were diagnosed and received therapies at earlier clinical stages (e.g., first or second LOTs), more effective and rational drugs or treatment approaches should be conducted to obtain a more profound response and a favorable survival outcome.

This study also explored the attrition rates by subgroup analysis of age, gender and ASCT. In this study, 88% of transplant MM patients received a second LOT and 13.7% received a fifth LOT, while only 50.9% of non-transplant received a second LOT and 3.3% received a fifth LOT. Similar to the results of the study by Fonseca et al. (2020), the attrition rates across all LOTs were relatively lower in transplant MM patients than in non-transplant MM patients. Besides, this study revealed a distinct difference in the attrition rates among MM patients of different ages. The attrition rates from 1st LOT through 5th LOT among MM patients ≥65 years old were generally higher than MM patients <65 years old, which may be explained by the different baseline conditions of these two groups of MM patients and elderly MM patients are usually frail and presented with more comorbidities (Lee et al., 2021; Tang et al., 2022). The median treatment duration was approximately six to 8 months across all LOTs, similar to other real-world study (Fonseca et al., 2020).

In this study, we performed the subgroup analysis of the baseline characteristics and comorbidities between MM patients receiving only 1 LOT and >1 LOT. MM who did not enter the subsequent LOT were significantly older, presented higher levels of β2-microglobulin and LDH, and had higher incidences of anemia, thrombocytopenia, renal insufficiency and comorbidities such as amyloidosis, pulmonary diseases, hepatic diseases, and cardiac disorders. It may indicate that younger MM patients may have the ability to receive more successive treatment, and a relatively preferable clinical status and physical state may be the main reason for receiving more subsequent therapies. We also noticed a significantly decreased proportion of ISS stage III MM patients in the group with >1 LOT. Especially we also conducted the Cox proportional hazards model to assess the risk factors for treatment attrition of the frontline therapy of MM in this study. Based on the multivariate analysis, ISS stage III, high LDH, and higher incidence comorbidities such as pulmonary diseases, hepatic diseases, and cardiac disorders were independent risk factors for treatment attrition of the frontline therapy. Overall, factors of older age, high-risk stratification, higher tumor burden, more comorbidities, and worse performance status may be associated with fewer subsequent treatments, increased drug toxicities and resistances.

Several limitations may exist in this retrospective real-world study. Firstly, it should be noted that this study only enrolled hospitalized MM patients who had received at least one LOT but excluded patients only receiving short-term glucocorticoids, which underestimates the attrition rates due to excluding untreated MM patients. Therefore, the results should be interpreted in the context of inpatients who received anti-tumor therapy, not the entire MM population. Secondly, patients did not enter a subsequent LOT generally owing to three reasons (i.e., death, loss of follow-up, or staying at the current LOT), but the data did not include information on the disease status of patients who survived at the current LOT during the study period. Besides, since this real-world study was carried out in a tertiary referral hospital in Western China, MM patients who lost follow-up in this hospital may return to local hospitals for further therapies, resulting in overestimated attrition. Thirdly, the shift of LOTs may be due to disease progress or drug intolerance, which was not deeply discussed in this study.

Collectively, this study demonstrated the common frontline treatment patterns in Chinese MM patients and elucidated the attrition rates with distinct LOTs and potential risk factors linked with treatment attrition, which may provide more evidence for effective treatments at specific LOTs in MM.

## Data Availability

The raw data supporting the conclusion of this article will be made available by the authors without undue reservation.
