# Supplementary material for: Treatment attrition rates and relevant risk factors in multiple myeloma: A real-world study in China
Source: Front Pharmacol. 2023 Jan 12;14:979111. doi: 10.3389/fphar.2023.979111 (PMC9877623; doi:10.3389/fphar.2023.979111)
Supplement: Supplementary file 1 [file Image1.PDF]

Supplementary Material

Supplementary Figures

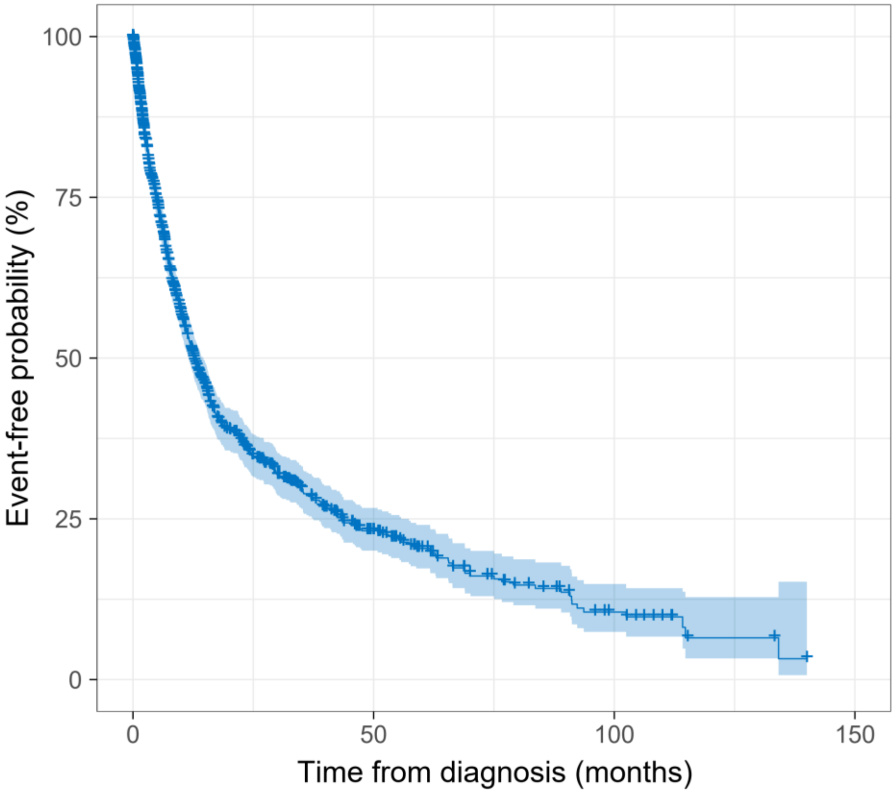

Supplementary Figure 1. Kaplan-Meier plot representation of the event-free probability of 1255 MM patients. The event was defined as the start of the second line of therapy.
